# Adolescent Mental Health: A Focus on Psychiatric Counseling from the Emergency Room of an Italian University Hospital in the Five Years from 2019 to 2023

**DOI:** 10.3390/ejihpe14050082

**Published:** 2024-05-05

**Authors:** Maria Giuseppina Petruzzelli, Lucia Marzulli, Giuseppe Colacicco, Fabio Tarantino, Flora Furente, Alessandra Gabellone, Lucia Margari, Emilia Matera

**Affiliations:** 1 Department of Translational Biomedicine and Neuroscience (DIBRAIN), University of Studies of Bari “Aldo Moro”, 70124 Bari, Italy; maria.petruzzelli@uniba.it (M.G.P.); g.colacicco14@studenti.uniba.it (G.C.); f.tarantino29@studenti.uniba.it (F.T.);; 2 Department of Precision and Regenerative Medicine and Ionian Area (DIMEPRE-J), University of Studies of Bari “Aldo Moro”, 70124 Bari, Italy; lucia.margari@uniba.it (L.M.); emilia.matera@uniba.it (E.M.)

**Keywords:** mental health, adolescence, self-harm, suicide

## Abstract

Recent studies have revealed increasingly worse and more complex mental health conditions in young people, which is reflected in a growing trend in emergency room (ER) visits for acute psychopathological symptoms (APSs). This phenomenon has become exacerbated in recent decades, with a peak in the post-pandemic period. To better characterize the phenomenon, we investigated the change in the rate and type of ER counseling requests provided at the Child Neuropsychiatry Unit of the University Hospital of Bari, Italy over the period between 2019 and 2023 for subjects younger than 18 years old. For this purpose, we retrospectively analyzed a total number of 1073 urgent consultation reports retrieved through the reporting computerized operating system of our hospital. The distribution of the counseling requests provided for APSs and, among these, the distribution of the numbers of APSs and of the male: female ratio were significantly different over the years, with an increasing linear trend identified for APSs (*p* = 3.095 × 10^−7^), the average number of APSs (*p* = 3.598 × 10^−7^), and female gender prevalence (*p* = 0.03908), as well as for the patients with a history of psychotropic drug assumption (*p* = 0.0006319). A significant change in the number of urgent counseling requests received for eating disorders (*p* = 0.0007408), depression (*p* = 7.92 × 10^−8^), somatization (*p* = 4.03 × 10^−6^), self-harm (SA) (*p* = 1.358 × 10^−6^), and non-suicidal self-injury (NSSI) (*p* = 8.965 × 10^−6^) was found, with a significant increasing trend for anxiety (*p* = 0.0444), depression (*p* = 8.06 × 10^−6^), somatization (*p* = 0.004616), SA (*p* = 3.998 × 10^−8^), and NSSI (*p* = 5.074 × 10^−7^). The findings of our study support the hypothesis of an alarming progressive worsening of the mental health of children and adolescents, with an overlapping effect of the pandemic exacerbating the process.

## 1. Introduction

Over the last fifteen years, a progressive increase in the prevalence of mental health problems in young people has been observed both in Italy and in the rest of the Western world, suggesting a breaking up of the link between cognitive and physical well-being, but the causes of this trend are not yet fully understood [1,2,3,4,5,6].

In such a scenario, the exceptional restrictive measures taken to contain the spread of SARS-CoV-2 infection, as well as fears of contagion and its consequences, hospitalizations, and deaths [7,8,9], ensured that the COVID-19 pandemic acted as a catalyst within this psychopathological vulnerability [10,11,12,13]. In addition, social interactions have suddenly changed quantitatively and qualitatively with an obligatory preference for virtual relationships; school closures, economic insecurity among families with lower socioeconomic statuses, altered sleep patterns with a reversal of circadian rhythms, decreased physical activity, unhealthy eating habits, and potential difficulties in family relationships, including exposure to intrafamilial violence/trauma, have heightened concerns about the emotional well-being of the adolescent population [12,14,15]. Admissions to the emergency room (ER) for acute psychopathology in young people during the first wave of the pandemic were down compared to the pre-pandemic period, likely due to the choice to stay at home out of fear of contracting COVID-19 in a hospital setting, social distancing and isolation, less travel, and the closure/limited activity of psychiatric care and rehabilitation centers [16,17,18]. At the beginning of the second wave of the COVID-19 pandemic, the prevalence rates of acute psychiatric disorders in children and adolescents exceeded pre-pandemic levels [19,20,21,22,23,24,25]. In Italy, the study group of the Child Neuropsychiatry Unit of the University Hospital of Bari conducted an epidemiological analysis of the children and adolescents referred for urgent consultation in the ER to estimate the prevalence rates of acute psychopathological admissions by comparing the period immediately before the start of the pandemic and the post-pandemic period, including the first and second waves of the COVID-19 pandemic [26]. This study found that ER visits for symptoms of depression, post-traumatic stress, psychosis, and self-harm in children and adolescents increased from 2019 to 2021. Subsequent studies conducted in other Italian hospitals confirmed these data [27], leading to the hypothesis that the persistence in time of the COVID-19 pandemic had a detrimental effect on youth psychological well-being, with plausible long-term implications.

On 5 May 2023, the general director of the World Health Organization (WHO), Tedros Adhanom Ghebreyesus, declared a public health emergency of international concern (PHEIC) due to the COVID-19 pandemic [28]. At the same time, in the hospital’s clinical practice, the increase in the number of requests for youth psychiatric emergencies continues to grow over time, suggesting that mental health crises in the child/adolescent population remain an important issue, irrespective of pandemic situations. The declining infection rates and widespread vaccination, along with the adjustments and adaptations taking place in the post-pandemic era, do not appear to be accompanied by a substantial reduction in adolescent psychiatric emergencies.

Beyond the increase in the number of adolescents who turn to the emergency room for psychopathological symptoms and regardless of the variability in the frequency of presentation of each clinical dimension, it seems like we are seeing a substantial change in the complexity of the psychiatric clinical presentations [29]. Compared to the pre-pandemic period, adolescents who reach acute psychopathological conditions present more and more complex aggregations of psychiatric symptoms, usually worsened by the risk of suicide behaviors. The co-occurrence and the relationship between the symptoms of depression, anxiety, and eating behaviors, complicated by self-harm and suicidal behaviors, makes both diagnostic assessments and therapeutic management more difficult [30]. Literature on the changing pattern of child and adolescent psychiatric emergencies between the pre- and post-pandemic era is rather limited. Thus, further longitudinal research is needed to better understand the long-term overall impact of the pandemic on adolescent mental health.

To explore the hypothesis of a quantitative and qualitative change in the clinical manifestations of psychiatric emergencies in children and adolescents, this study aimed to investigate the requests for psychiatric consultations from the ER of the University Hospital of Bari, Italy, in people under 18 years of age, comparing the periods between 2019 and 2023. We focused on the following: (1) the changes over time in the rate of requests for acute psychopathological symptoms (APSs) on the total counseling requests from the ER; (2) the changes over time in the number of consultancy requests divided by the socio-demographic characteristics of the patients; (3) the changes over time in the types of psychopathological symptoms motivating the presentation to the ER with a focus on self-harm behaviors; (4) the changes over time of the co-occurrence of multiple psychopathological symptoms in adolescent patients presenting to the ER.

## 2. Materials and Methods

For this study, we retrospectively analyzed reports of urgent specialist counseling from the ER of the University Hospital of Bari for the onset of APSs (anxiety, agitation, eating disorders, depression, somatization, trauma, psychosis, drug abuse, and self-harm) for children and adolescents up to 18 years of age and provided at the Child Neuropsychiatry Unit of the same hospital. One parent/legal guardian was always present during the counseling.

We included all the requests for consultation from the central ER, the ophthalmological ER, and the ER of the “Giovanni XXIII” Children’s Hospital. Moreover, non-urgent consultation requests from other departments were excluded.

To avoid a bias in the data collection due to the decrease in the number of hospital admissions for mental health reasons in the period of the first lockdown (March–May 2020), we selected and compared all urgent consultations that were carried out in the following time periods:

- July to December 2019 (referred to as “2019”)
- July to December 2020 (referred to as “2020”)
- July to December 2021 (referred to as “2021”)
- July to December 2022 (referred to as “2022”)
- July to December 2023 (referred to as “2023”).

Requests for which the data or consultation reports were missing were excluded.

The computerized operating system “Galileo” was used to search for the consultation requests and corresponding reports. This operating system collects information on the identification, reception, and clinical and diagnostic procedures of patients who contact our hospital; the data, which can be accessed by any authorized healthcare worker with their credentials, is shared by all the hospital’s outpatient departments, ensuring fully integrated management.

We collected data on age, gender, previous medication intake, reasons for ED access, and symptoms observed during the consultation. Based on the information in the consultation report, members of the research group independently assigned the reported symptoms to non-psychiatric urgency/emergency, generally referring to neurological symptoms (e.g., epilepsy, migraine, multiple sclerosis, head trauma, etc.) or, inversely, to one of the following psychopathological dimensions (hereafter referred to as acute psychopathological symptomatology or APSs): anxiety, psychomotor agitation, eating disorders, depression, somatization, psychological trauma, psychosis, substance abuse, and self-injuring behaviors (self-harm) for both suicide attempts (SA) and non-suicidal self-injury (NSSI). Any discrepancies were discussed and resolved under the supervision of a senior researcher. The study was approved by the local independent ethics committee of the University Hospital of Bari (PS-C19).

### Statistical Analysis

All variables were collected in a structured form specific to this research. Continuous variables were reported as means and standard deviations and as medians and interquartile ranges, while categorical variables were expressed in numbers and percentages.

Significant differences in the medians of age and numbers of APSs (whose normality of distribution was tested using the Shapiro–Wilk test) were analyzed using a Kruskal–Wallis test. The Jonckheere–Terpstra trend test for ordered alternatives was used to evaluate the existence of an increasing trend.

For the categorical data related to the total number of consultations and partial numbers of consultations divided according to the reasons for access, the independence of proportions was tested using the Pearson-X2 test. Finally, the Mantel–Haenszel linear-by-linear association Chi-squared test was used to verify the presence of a linear trend in the proportions.

Descriptive and statistical analyses were performed in the statistical environment R version 4.1.2 [26,29] (The R Foundation for Statistical Computing; Vienna, Austria); the calculations of the means, standard deviations, medians, ranges, and interquartile range of the samples were performed using Excel statistical functions. The statistical significance was set with an alpha of 0.05.

## 3. Results

Table 1 and Table 2 summarize the socio-demographic and clinical data from this retrospective data collection. A total number of one-thousand and seventy-three consultation requests were analyzed; six of these were excluded due to missing reports, with the remaining number of one-thousand and sixty-seven analyzed consultations.

A significant difference emerged between the proportions of consultation requests provided for APSs and the total requests (*p* = 3.596 × 10^−8^), with a significantly increasing linear trend over time (*p* = 3.095 × 10^−7^).

Among the patients referred for APSs, the average age of the sample did not differ significantly between the groups according to what emerged from the Kruskal–Wallis test, nor was there an increasing trend in the median age from the Jonckheere–Terpstra test. Conversely, a significant difference in the average number of APSs emerged from the Kruskal–Wallis test between the years, with a significantly increasing trend from 2019 to 2023. A prevalence of the female gender emerged, significantly variable over the years (*p* = 0.04014), with an increasing trend (*p* = 0.03908); furthermore, a significant variation over the years (*p* = 2.716 × 10^−5^), with an increasing trend (*p* = 0.0006319) emerged for the proportion of patients with a positive medical history due to previous use of psychotropic drugs.

Significant differences emerged in the proportions of urgent consultation requests received for eating disorders (*p* = 0.0007408), depression (*p* = 7.92 × 10^−8^), somatization (*p* = 4.03 × 10^−6^), self-harm (*p* = 1.358 × 10^−6^), and NSSI (*p* = 8.965 × 10^−6^), with a significant increasing trend for anxiety (*p* = 0.0444), depression (*p* = 8.06 × 10^−6^), somatization (*p* = 0.004616), self-harm (*p* = 3.998 × 10^−8^), and NSSI (*p* = 5.074 × 10^−7^) (see Table 3).

## 4. Discussion

The main findings of this study were that between 2019 and 2023, there was a significant upward trend in the proportion of consultation requests for APSs, especially adolescents of the female gender and with previous use of psychotropic drugs. A significant increasing trend over time emerged in the proportions of consultation requests for anxiety, depression, somatization, and self-harm. However, of greater significance is the increasing trend of the number of co-occurring different psychopathological symptoms.

The reasons for this increase over time are various, partly still unknown, and for this reason, they still need to be sought and studied to implement timely and additional primary and secondary prevention strategies to better deal with the post-pandemic setting and new emergencies.

The increasing trend in ER consultations for APSs could be justified by the rebound effect related to the return to normal daily activities due to the suspension of the lockdown phase. In fact, during the first lockdown, young people may have experienced transitory relief from some aspects related to schools, such as school refusal, stressful educational demands from parents and teachers, learning difficulties, and tensions relating to academic performance and bullying victimization, but it seems less likely that these justifications could motivate the maintenance of such trend in subsequent years [26,31].

If the second lockdown period was characterized in Italy by the gradual resumption of some work occupations but by limitations on the schools’ activities with the prosecution of online lessons, the subsequent reopening of schools with the restart of demands for performance and competition among peers may have favored such increase [32]. Contemporary changes in social and relational dynamics, linked or not to the role played by new technologies, may have diminished the family authority and contributed to the contemporary inability of parents to manage adolescents on an emotional, normative, and psychopathological level [33]. In child neuropsychiatry outpatient services, there could be challenges due to limited resources and organizational gaps in addressing the growing prevalence of adolescent psychopathology [34].

The frequency distribution of consultancy requests for adolescents and females, especially for those with prior poor mental health, showed a significant increasing trend over time, inevitably accentuating the introduction of pharmacological therapy and intensifying a trend already developed in previous years [35]. The increase in adolescent psychopathology is crucial because it compromises the social and relational functioning of young people with possible consequences on personality development, academic and work careers, and mental and physical health in adulthood [36]. Age-specific features may expose adolescents to a greater risk of mental health difficulties than the adult population due to physical development and brain chemical and structural changes (i.e., amygdala circuits, white matter microstructures in the uncinate fasciculus, cingulum, bilateral longitudinal fasciculi, anterior thalamic radiation, callosal body, and corticospinal tract) that intensify emotional reactions to stress-inducing factors, as well as slower development of the emotional self-regulation system and increased social sensitivity [37,38]. Compared to children, adolescents can engage in destructive and self-harming behaviors, which complicate the organization of outpatient treatment [36].

Females, representing the largest diagnostic group in our sample, in particular, are generally more exposed to a greater vulnerability to the development of internalizing and stress-related psychiatric disorders, considering the sex-specific hormonal differences in the estrogen–testosterone ratio and the variability in the timing of physical development and changes associated with menarche and menstruation through the modulation of the neuroendocrine system [37,38]. Social and environmental factors in contemporary society, such as the high prevalence of sexual abuse, machismo, the acceptance of violence against women, stalking, sexting, prostitution [39,40], coping with the burden of multiple social roles, differences in social norms and support, and the contexts in which females grow up and are socialized in, alongside the disparities in cultural norms and behaviors [41] and the fact that daily medical practice is still far away from being sensitive to sex [42] could explain the high prevalence of female patients in our sample.

In addition, the growing share of patients already being treated with psychotropic drugs and known to specialist health services supports the hypothesis that adolescents with psychiatric pathologies due to a bio-psychological vulnerability would be particularly at risk of reporting negative effects related to the difficulty of coping with additional stressful and prolonged factors [43,44].

Between 2019 and 2023, a significant difference in the proportions of anxiety, depression, eating disorders, somatization, and self-harm was recorded, with an upward trend over time. Moreover, the increase in the rate of adolescent patients presenting to the ER with the co-occurrence of multiple psychopathological symptoms requires further efforts to elucidate the underlying factors that lead to acute crises and to design effective strategies for prevention and early intervention. If we focus on self-harming, which often complicates and accompanies the above psychopathological problems, including depression and eating disorders, the number of NSSIs compared to SA progressively increases. Suicidality and self-harm are phenomena severely impacting the young population and closely interrelated: self-injuring behaviors are risk factors for SA, and previous episodes of self-injuring behaviors and suicidality are predictors of further self-harming behaviors over time [45,46,47]. Some authors have reported that a certain part of the SA among young people can probably be interpreted as a form of non-suicidal self-harm, especially if it occurs through self-poisoning, which usually has a lower lethality than adults and is considered related to a lower suicidal intent than other suicidal methods. So, the increase in NSSI over time could probably be even more significant, and many forms of SA, as well as NSSI in youth, could be considered as an attempt at self-medication and/or dysfunctional emotional self-regulation [48]. Emotional difficulties are experienced, especially in adolescence, with an emphasis on the body for difficulties in encountering one’s emotions on both a physical and psychological level [49]. The relationship with body image is part of the determinants of self-harming behavior, not just emotional dysregulation, and like emotional dysregulation, it acts as a transdiagnostic factor of vulnerability for multiple psychiatric disorders. This is of considerable clinical importance and requires certain therapeutic attention, especially in adolescence, a period in which the evolutionary process of identity formation is intricately linked with the importance attributed to body image, a multidimensional construct essential in the formation of one’s self-concept and the extent to which one is concerned about deviations from internalized body ideals [50].

Regardless of the reasons that could explain the significant increase in the symptoms of anxiety, depression, eating disorders, self-harm, and somatization motivating presentations to the ER, we could support the hypothesis that the time trends of APSs could reflect the trend over the last century in the worsening of the mental health of children and adolescents, with an overlapping effect of the pandemic, which could have created a new social and health scenario and worsened the state of the mental health of the young population [27]. Certainly, at present, greater clinical severity and etiopathogenic complexities require less immediate and more complicated treatments than in the past. Such a course could be partly influenced by the fact that a doctor is only consulted when a health problem has already worsened and that multiple consultations are sought, especially when there are discrepancies between medical opinions [51], which makes further treatment more difficult. During the COVID-19 pandemic, a delay in consulting a doctor was associated with fear of infection, COVID-19 information awareness, and fact-checking amid the pandemic, especially among females and younger age groups [52].

In addition, the years 2020 and 2021, with their different measures to contain the pandemic activated in Italy from 9 March 2020 to 31 March 2022 [53,54,55,56,57], may have contributed to the observed differences in the mean scores. More specifically, in 2020, social distancing and isolation, the reduction in travel, and the decision of sick people to stay at home for fear of contagion, as well as the reduced availability of outpatient medical care for various disease treatments (e.g., via video call) could explain the decrease in visits to the ER [58]. Their subsequent increase in 2020 and in the following years, when outpatient specialist care was gradually restored, supports the hypothesis that the persistence of the COVID-19 pandemic had a long-term impact on the mental well-being of adolescents [26]. Limitations of this study include the small sample size and retrospective data collection via information systems that have a clinical rather than research purpose. Since the data were collected using computer systems structured for first-aid consultations, their accuracies are partial. For these reasons, there is no information relating to the risk factors for mental illness (family conflicts, familiarity, and socio-economic situation) and the clinical course of acute psychiatric disease.

The data from this study should be considered preliminary.

## 5. Conclusions

In conclusion, our data showed a significant increase over time in the proportion of ER consultation requests for APSs, in particular for adolescents, females, and young people with previous use of psychotropic drugs. Moreover, we observed an increase in the rate of adolescents referring to the ER, with a higher co-occurrence of different psychopathological symptoms. Anxiety, eating disorders, depression, somatization, and self-harm also exhibited a significant increase between 2019 and 2023, justifying the current need to recognize the mental health of young people as an emergency at both a social (e.g., familial, scholastic, recreational, and religious) and health (e.g., training of healthcare staff) level.

In terms of practical implications, neuropsychiatric services should urgently adapt their organization and resources for the early recognition and treatment of acute psychiatric problems to avoid the chronic course of the disease and its negative existential outcomes [59]. This suggests the need for a greater overall number of beds and expert doctors trained specifically for the management and treatment of new youth psychiatric emergencies. Family and parenting support interventions should also be considered of strategic importance to reduce negative outcomes, just as correct post-discharge management through collaboration with extra-hospital child neuropsychiatry services. Suicide prevention interventions, both individual and social, should be adopted.

This longitudinal research could be continued, preferably through collaboration with other national and international hospital and community neuropsychiatry units. It will, first of all, be necessary to conduct a collection of data that seeks to overcome the limitations of this study (e.g., comparing periods that do not include the COVID-19 pandemic and its consequent containment measures) and evaluate the analysis of other information, including the level of education, environmental and family risk factors, the subsequent course of APSs, and possible hospitalization, which could help for a better understanding of these phenomena.

## Figures and Tables

**Table 1:** Number of requests for APSs during the years of 2019–2023.

	2019 *n* = 246	2020 * *n* = 178	2021 ** *n* = 225	2022 *n* = 199	2023 *n* = 225	Pearson-X2	*p*-Value	Mantel–Haenszel-X2	*p*-Value
**APS requests**	125	96	159	148	151	40.389	**3.596 × 10^−8^**	26.189	**3.095 × 10^−7^**

* Living with COVID-19 (until 7 October 2020), new restrictive measures (8 October–5 November 2020), differentiated containment measures with respect to the levels of contagion in Italian regions (6 November–20 December 2020), measures for the Christmas holidays (from December 2020). ** Easing of containment measures (until 5 August 2021), vaccination green pass (from 6 December 2021).

**Table 2:** Socio-demographic and clinical features of patients referred for APSs.

	2019 *n* = 125		2020 * *n* = 96		2021 ** *n* = 159		2022 *n* = 148		2023 *n* = 151					
	Mean (SD)	Median (IQR)	Mean (SD)	Median (IQR)	Mean (SD)	Median (IQR)	Mean (SD)	Median (IQR)	Mean (SD)	Median (IQR)	Kruskal–Wallis Test	*p*-Value	Jonkheere–Terpstra Test	*p*-Value
**Age**	13.3 (3.6)	14.0 (5)	12.8 (4.1)	14.0 (6)	14.0 (3.4)	15.0 (4)	13.5 (3.6)	15.0 (4)	13.95 (3.2)	15.0 (3)	7.3162	0.1201	95373	0.09292
**Number of APSs**	1.34 (0.6)	1	1.26 (0.49)	1	1.45 (0.71)	1	1.84 (0.99)	2	1.79 (1.05)	1	43.224	9.299 × 10^−9^	112396	3.598 × 10^−7^
	***n***		***n***		***n***		***n***		***n***		**Pearson-X2**	***p*** **-value**	**Mantel–Haenszel-X2**	***p*** **-value**
**Female gender**	56		46		98		81		85		10.017	**0.04014**	4.2573	**0.03908**
**Previous psychotropic drugs**	32		31		79		50		73		26.329	**2.716 × 10^−5^**	11.68	**0.0006319**

* Living with COVID-19 (until 7 October 2020), new restrictive measures (8 October–5 November 2020), differentiated containment measures with respect to the levels of contagion in Italian regions (6 November–20 December 2020), measures for the Christmas holidays (from December 2020). ** Easing of containment measures (until 5 August 2021), vaccination green pass (from 6 December 2021).

**Table 3:** Results of statistical comparisons of the number of urgent consultation requests for different APSs (Pearson X-squared test) and analysis of trends (Mantel–Haenszel X-squared test).

	2019	2020 *	2021 **	2022	2023				
APS Requests (*n*)	*n* = 125	*n* = 96	*n* = 159	*n* = 148	*n* = 151	Pearson-X2	*p*-Value	Mantel–Haenszel-X2	*p*-Value
**Anxiety (*n*)**	53	30	57	42	47	7.2228	0.1246	4.0399	**0.04444**
**Agitation (*n*)**	48	39	71	67	66	1.8982	0.7545	1.2159	0.2702
**Eating disorders (*n*)**	15	4	16	32	16	19.209	**0.0007151**	2.1257	0.1448
**Depression (*n*)**	19	2	24	46	29	15.321	**0.00408**	4.4837	**0.03422**
**Somatization (*n*)**	12	20	13	1	9	32.731	**1.356 × 10^−6^**	11.208	**0.0008147**
**Trauma (*n*)**	6	10	5	9	14	7.8391	0.09765	0.86097	0.3535
**Psychosis (*n*)**	5	7	19	11	11	6.4509	0.1679	0.66322	0.4154
**Drug abuse (*n*)**	2	4	2	3	9	8.0957	0.08814	2.6117	0.1061
**Self-harm behaviors (*n*)**	13	9	30	35	46	25.998	**3.168 × 10^−5^**	24.454	**7.612 × 10^−7^**
**SA (*n*)**	6	5	15	13	16	4.6438	0.3258	3.9029	**0.0482**
**NSSI (*n*)**	7	4	15	22	30	22.07	**0.0001941**	20.005	**7.725 × 10^−6^**

* Living with COVID-19 (until 7 October 2020), new restrictive measures (8 October–5 November 2020), differentiated containment measures with respect to the levels of contagion in Italian regions (6 November–20 December 2020), measures for the Christmas holidays (from December 2020). ** Easing of containment measures (until 5 August 2021), vaccination green pass (from 6 December 2021).

## Data Availability

The data presented in this study are available on request from the corresponding author.
